# The role of community champions in place-based early years support: how can we successfully share knowledge and build parent confidence?

**DOI:** 10.1177/17579139231203181

**Published:** 2024-05-17

**Authors:** SB Mitchell, G Marks, J Lloyd

**Affiliations:** Children & Young people’s Mental Health Research Collaboration, University of Exeter Medical School, South Cloisters, St Luke’s Campus, Exeter, EX1 2LU, UK; Action for Children, UK; Medical School, University of Exeter, Exeter, UK

## Introduction

A child’s early life experiences and the relationship they have with their caregiver significantly influences the trajectory of their cognitive, emotional, behavioural and social development across the life course.^1^ Supporting parents in their caregiving role during a child’s early years, particularly those parents from low-income families, is crucial in addressing health inequalities.^2^ Children from low-income families are at particular risk of delay and impairment and are more likely to have poorer social and emotional wellbeing than their peers.^3^

Research has demonstrated the critical role that Early Year’s intervention services can play in reducing health inequalities^2^; however, there is differential reach in the uptake of parenting programmes with poorer attendance for low-income parents, suggesting that those parents with the greatest potential to benefit may be the least likely to engage.^4^ There is, therefore, a need for a new approach; one that is socially sensitive, trusted and sustainable and, crucially, able to engage parents across the socio-economic spectrum.

Research evidence supports the need for more community-centred approaches to health and wellbeing,^5^ particularly for disadvantaged families, and involving volunteers as community assets has been highlighted as a key strategy.^6,7^ In particular, existing work highlights the importance of trust and relationships when it comes to successful implementation of these programmes.^8^ While more research is needed, the impact of community champions on behaviour is promising, such as increased accessing of services, positive lifestyle changes and improved self-management of conditions such as diabetes.^9^ In this article, we present a case study of a community programme using volunteer champions to show how trust and relationships were developed with parents.

## Case Study: Building Babies Brains

Run by the charity Action for Children in Devon Children’s Centres, the programme’s aim is to take evidence-based neuroscience, historically held by ‘professionals’, and make the information and associated parenting strategies accessible to parents within the community, using community champions to disseminate this knowledge as part of their everyday interactions. Champions, working across four diverse communities across Devon, were trained to ‘bridge the gap’ between parents and professionals by offering reassurance, building trust, disseminating messages and signposting to professionals if needed.

**Figure:**
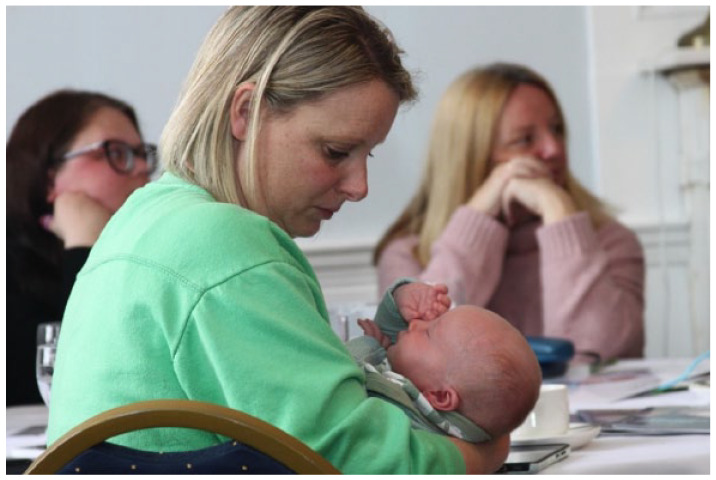
credit image to Action for Children

Action for Children commissioned the University of Exeter to complete interviews with 15 community champions. Ethical approval was obtained through Devon Children’s Centres, and consent was sought from each participant prior to interview. Interviews explored community champion motivations and experiences in relation to training, support and dissemination of messages. Interviews were analysed thematically using a deductive approach.^10^ This article will share core relational elements that led to improved peer-to-peer knowledge exchange.

## Creating a Receptive Context for Building Trusted Relationships

**Figure 1:**
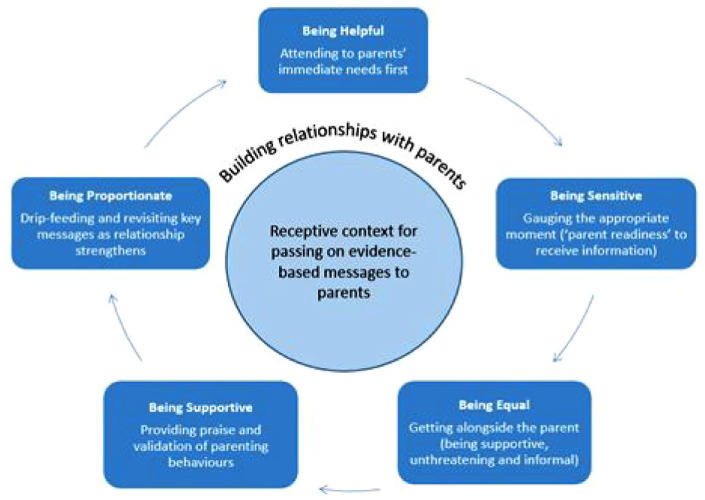
Relational elements supporting peer-to-peer knowledge transfer Source: Lloyd, Mitchell & Marks, 2020.

Champions described both successes and challenges in passing messages on in their community. In terms of successful experiences, champions described how their ability to pass messages on was supported by creating a receptive context which involved building relationships with parents through a series of phases: attending to the parents’ immediate needs, gauging the right moment, getting alongside the parent, providing praise and validation, and revisiting (see Figure 1). The process of building relationships followed a similar pattern across champions. While some champions described instances of passing on messages to strangers, successful interactions were largely described as being set within the context of a friendly relationship in a setting where the parent could be revisited. Champions emphasised the importance of the relational aspect of their role, building and growing a network within which messages could be passed on.

### Attending to immediate needs

Preparing the parent was seen as the first part of the process. Offering help was often used to initiate a relationship with a parent by attending to what’s needed in the moment (e.g. providing practical help) and then waiting to revisit that individual on another occasion, after building the relationship further when they might be more receptive or more able to take on information.

### ‘Gauging the moment’

Interactions were described as most challenging when family members offered conflicting and/or outdated advice and when approaching someone with a fixed mindset. Readiness to receive information was something mentioned by several champions as an important consideration when attempting to pass on messages to parents. In this situation, they acknowledged that sometimes other things needed to be attended to in the moment or that the parent simply was not yet ready and the focus needed to change to ‘preparing the parent’ and building a relationship.

Establishing a relationship with a parent prior to passing on knowledge was seen as key to successful interactions, although knowing exactly when to take the opportunity to ‘share’ was challenging. Champions described ‘gauging the moment’ and using non-verbal cues to inform their decision. Champions described a very gentle approach, testing the water to gauge parent receptiveness: *‘You can always smile and say, “Hi”, or, “Oh it looks like you are having a full-on day”, or something and see how the waters lie. I mean, some people give off a very clear message that it isn’t the right time to talk to them, they’ve got more than enough on their plate. So yes, I think you do have to gauge it’* (p. 19).

### ‘Getting alongside’

In line with perceptions of the role of a community champion as bridging the gap between professionals and parents, champions described ‘being alongside’ the parent as an important strategy when they began to interact. This encompassed a variety of approaches including using empathy, qualifying statements, and being unthreatening and informal.

Offering empathy or sympathy were described as fundamental in terms of initiating a caring interaction; being sensitive to what the parent is struggling with, normalising it and getting alongside. Being supportive, unthreatening and interacting in an informal way was also highlighted as important for successful exchanges.

Champions stressed the importance of avoiding trying to ‘rescue’ people’s parenting and ensuring that you could intervene without making the parent feel inadequate: *‘If it’s done appropriately, it’s non-threatening, and it’s just about that support really. It’s very supportive, there’s lots of information, but it’s all supportive and none of it is critical, and I think that is really important’* (p. 1).

### Praise and validation

Offering praise and validation was described as something which could help to cement and build relationships further. One champion described this as providing a *‘magnifying glass to focus on the good stuff’* (p. 13).

### Revisiting

A common approach was to drip feed information over time as the relationship was built. Settings that provided an opportunity for regular contact, such as a toddler group, were viewed as having the most potential for knowledge exchange. Not only do they provide opportunities to build on, repeat and reiterate key messages but also to strengthen relationships – essential for parent engagement.

## Conclusion

In this article, we present what helped community champions share child development knowledge so that it was accepted and understood.

Champions described the importance of building trusting relationships through a series of phases to create the context in which messages could be successfully transferred. A relational approach was seen as key to success, particularly for those parents most in need of support. Further research is needed to explore the reach of these messages within a community and the extent to which they impact parent behaviour.

## Supplemental Material

sj-docx-1-rsh-10.1177_17579139231203181 – Supplemental material for The role of community champions in place-based early years support: how can we successfully share knowledge and build parent confidence? Supplemental material, sj-docx-1-rsh-10.1177_17579139231203181 for The role of community champions in place-based early years support: how can we successfully share knowledge and build parent confidence? by SB Mitchell, G Marks and J Lloyd in Perspectives in Public Health
