# Supplementary material for: The role of community champions in place-based early years support: how can we successfully share knowledge and build parent confidence?
Source: Perspect Public Health. 2024 May 17;144(3):143–5. doi: 10.1177/17579139231203181 (PMC11103910; doi:10.1177/17579139231203181)
Supplement: sj-docx-1-rsh-10.1177_17579139231203181 – Supplemental material for The role of community champions in place-based early years support: how can we successfully share knowledge and build parent confidence? [file sj-docx-1-rsh-10.1177_17579139231203181.docx]

**Supplementary Table 1. Creating a receptive context**

| Sub-theme | Selected quotations |
| --- | --- |
| Attending to immediate needs | “…a mum that was more sort of struggling to listen, she was too kind of het up with how her child was being, so it was just for me to go back and relook at the situation and calm her down before I’d start giving her all this information. So I learnt that actually the child side of it, leave it, get her back into a safe thinking, make sure she’s okay and then fit the child’s thing in after” (P12).  “Well, if I’m approaching someone, then it’s probably that something’s either just gone wrong, or they’re struggling. So, I would actually see if there’s something practical I can do to help in that situation, so offer them some practical help. “Oh, can I hold your bag for you?” “Would you like me to fetch the buggy for you?” “Is there anything I can do to help?” type of thing. Something that I could see that actually, if somebody did that for me, that would be useful” (P1). |
| Gauging the moment | “I don’t think you can just dive in. It is a conversation, it’s not something that you can push on people” (P1).  “I think you have to do it on the families’ terms rather than your agenda” (P14)  “…to bridge that gap and to actually give her advice has been more tricky…there’s a long game, I suppose, to play with some people… they’ve got to almost be on their own journey and recognise… It’s really tricky, it’s such a personal thing. You’ve got to seek out that information, or be wanting to receive it. And I do think there’s a risk to it that you could put people off it by coming on too strong, or by just making it seem too much and too overwhelming” (P3).  “I think that’s one of the tricky parts, knowing when is the right time, when someone is going to be receptive to it because you can perhaps… I guess listening out for some kind of indicator that somethings is not working or they are struggling and thinking ooh I know something that might be able to help with this and then, yes, trying to bring it into the conversation without sounding like I’m telling them what to do, making suggestions and things (P16) “I think by talking to someone and just judging the situation, how open someone is to talk to you and how their response is when you are starting to stay things. If someone starts to shut you down straightaway there’s no point in carrying on” (P5).  “…just gently check in with whatever issue they have, how it’s going, and then see if they’re receptive to it or not really. It’s a very gently, gently approach” (P3)  “You can always smile and say, “Hi,” or, “Oh it looks like you are having a full-on day,” or something and see how the waters lie. I mean, some people give off a very clear message that it isn’t the right time to talk to them, they’ve got more than enough on their plate. So yes, I think you do have to gauge it” (P19).  “I think one of the biggest challenges I found with mums that I’ve been helping has been family members, so they’ll ask me questions and I’ll talk to them and then they’ll come back and they’ll say, ‘Well I was doing this, but then my mother-in-law said that I shouldn’t be because I will just spoil my child, I’m going to spoil my child’ and I think that’s a real… big barrier because it does kind of I suppose put a bit of a block there” (P4)  “It doesn’t work so well when people already have a fixed mindset. So, if they have their own attachment issues and difficulties and they have a fixed mindset, you really struggle to get them to accept the information that you’re giving them and be willing to put any effort into changing” (P1). |
| Getting alongside | “My approach is quite gentle, I suppose. I would always just sort of think that would just be my approach anyway, to come at it from a place of non-judgement and trying to be alongside people, trying to walk alongside people and allow them to tell their story” (P19)  “I think that’s a key thing really, that you don’t want to set yourself up as any kind of an expert, it’s much more of being alongside and, ‘Oh I’ve been through that, I had that happen,’ or, ‘My friend has this going on with her little one’. Just friendly really” (P8).  “The whole thing is just about empathy, isn’t it? You need to be going up to somebody in a manner that you are subservient and not dominant, really…And then just from there, just say, ‘Oh gosh, it’s tough on days like this, isn’t it? I remember my own toddlers’ and just about taking that stance of sympathy as quickly as you can. So, usefulness, sympathy, and then from there see if you get an opening” (P1).  “I often use my failings as examples, so I’ll often say, “I find this really hard because I’ve no idea how to do it, but what I’ve found helpful is this”(P13).  “I think it helps when it’s quite informal, it’s just a chat, a friendly chat. Yes, I think it’s completely unthreatening. They can take no notice whatsoever, can’t they, if they feel like that” (P8)  “I think where people are open, it works really well. It’s non-threatening. If it’s done appropriately, it’s non-threatening, and it’s just about that support really. It’s very supportive, there’s lots of information, but it’s all supportive and none of it is critical, and I think that is really important” (P1)  “I think probably the biggest thing, which I very much feel anyway, is to kind of let parents off the hook in a way, to be the voice that’s saying there is no judgement here, I’m a safe person that you can kind of just chat to and actually we all struggle and it very much reinforced that kind of way” (P19) |
| Praise and validation | “Often, when mums are being really harried, I’ll try and make the effort to validate their job and their role and sort of say, ‘Gosh, you are doing such a great job. It’s the hardest job in the world, isn’t it, being a mum?’ ‘…Sort of validating what they are doing. And if I’ve seen good stuff, kind of saying that as well, sort of saying, ‘Oh gosh, you were so great and calm. I would never feel like that. That’s so helpful because even though they are having a tantrum in the middle of the supermarket, you are so great at just meeting their needs there and then’. So yes, I think it’s acknowledging their hard work and using a magnifying glass to focus on the good stuff.” (P13). |
| Revisiting | “…well I see them regularly, you see, so yes, I do revisit that. But what’s really interesting is if people really want to know more, they revisit it with you” (P1)  “…it would be a number of chats over time and she would come back and say, “Oh that really helped,” or, “This is what’s happening now,” and I think it’s almost a listening ear. I think the other really valuable thing is that when you give people time, and I guess using the five concepts of… Just getting alongside the families and validating their feelings, that provides a really good basis of empathy, doesn’t it, so that you’ve developed a relationship so when they see you again, that will trigger that oh that was a really useful conversation that I had and feeling open to be able to have these conversations straight again, so you almost pick up from where you left off with” (P13)  “I’ve got people that I might have seen over a few weeks at different things and I suppose I try to clock it a little bit if I see somebody and I think I’ve tried to interact and they are just not in the place, so I think oh maybe next week, because I know I’m going to see them at x, maybe next week when things are a bit calmer I might try and remember that and come alongside them. So I think that approach has worked quite well… o I think it’s not like you’ve got to get across all these things in one go. I think you build up a picture. And relational stuff is always kind of the stuff that works much better in my experience” (P19) |
